# 961. Pandemic Hits: Evaluation of an Antimicrobial Stewardship Program Website for Hospital Communication During the COVID-19 Pandemic

**DOI:** 10.1093/ofid/ofac492.804

**Published:** 2022-12-15

**Authors:** Reinaldo Perez, Michael E Yarrington, Rebekah Wrenn, Connor R Deri, Martha B Adams, Richard H Drew, Rebekah W Moehring, Michael J Smith, Justin Spivey

**Affiliations:** Duke University, Durham, North Carolina; Duke University Health System, Durham, North Carolina; Duke University, Durham, North Carolina; Duke University, Durham, North Carolina; Duke University, Durham, North Carolina; Duke School of Medicine/Campbell University College of Pharmacy & Health Sciences, Durham, North Carolina; Duke University, Durham, North Carolina; Duke University, Durham, North Carolina; Duke University, Durham, North Carolina

## Abstract

**Background:**

Antibiotic Stewardship Programs (ASPs) assist front-line clinicians in synthesizing emerging data and establishing best practices. Our ASP team directly maintained and edited an internal web application, Duke CustomID^®^, to disseminate updated guideline, policy, and drug information during COVID-19. We aimed to describe website engagement and maintenance during the dynamic pandemic period.

**Methods:**

We performed a descriptive, time-series analysis using Google Analytics software to measure engagement with Duke CustomID^®^ during a 1-year pre-pandemic period through the Omicron surge: January 2019 to March 2022. We measured total page views (or “hits”), COVID-specific page hits, and days requiring COVID-specific page edits by week. Given fluctuations in hospitalization rates, we defined the primary outcome as the rate of hits divided by total hospitalizations. Weekly data were assessed graphically with positive COVID tests and COVID hospitalizations. We used negative binomial regression to quantify the association between COVID hospitalizations and hit rates and to trend engagement over time, adjusted for seasonality. We stratified data by COVID page and calculated a hit/edit ratio.

**Results:**

Engagement with CustomID® increased during the pandemic period, especially during surges (**Figure**). Hits in the pre-pandemic period were median 1707 (range 1165-2354) per week, and hit rates median 1.95 per hospitalization (range 1.40-2.86). Peaks were observed in March 2020 (hit rate 4.59) and January 2022 (hit rate 3.87). On average, for every 100 COVID hospitalizations, the hit rate increased by 0.08 (0.004-0.16, p=0.04). Engagement slowly increased over the study period (relative rate week 1 versus 170: 1.15, 95% confidence interval 1.02-1.28, p=0.02). COVID page edits per week had a median of 2 (range 0-12). Adult Inpatient Guidelines and COVID Monoclonal Antibody pages had highest use (**Table**).

Duke CustomID Hits and Maintenance Efforts over the Pandemic

**Figure:**
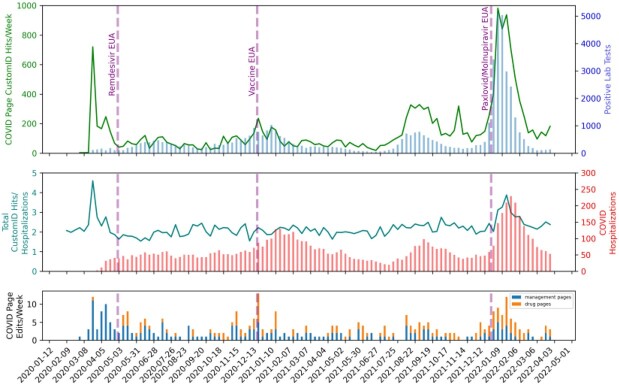

Top: COVID-specific CustomID hits per week (Green), Positive COVID tests per week (Blue) over time

Middle: Total custom ID page hits relative to total hospitalizations per week (teal), COVID hospitalizations (Red)

Bottom: Number of edits to COVID-specific CustomID pages per week, stratified by management pages and drug pages

Several dates of significance are highlighted including the Emergency Use Authorizations (EUA) for remdesivir, the COVID Vaccines, and Paxlovid

Duke CustomID COVID-19 Page Hits and Edits

**Figure:**
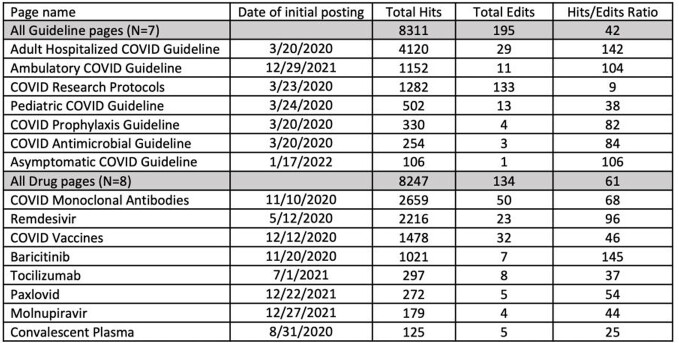

COVID specific pages on Duke CustomID with total hits, edits, and ratio over the pandemic

**Conclusion:**

Our ASP’s website was a highly utilized, practical tool for disseminating practice-changing information during the pandemic. Use increased over time and especially during surges. An electronic reference customized for local practice and rapidly updated by ASPs offers critical support for front-line clinicians.

**Disclosures:**

**Martha B. Adams, M.D.**, Custom Clinical Decision Support, Inc: Board Member|Custom Clinical Decision Support, Inc: Ownership Interest **Richard H. Drew, PharmD MS**, American College of Clinical Pharmacists: Publication royalties|Takeda: Advisor/Consultant|UpToDate: publication royalties **Rebekah W. Moehring, MD, MPH, FIDSA, FSHEA**, UpToDate, Inc.: Author Royalties **Michael J. Smith, M.D., M.S.C.E**, Merck: Grant/Research Support|Pfizer: Grant/Research Support.

